# Swallowing disorders in patients with obstructive sleep apnea: a critical literature review

**DOI:** 10.5935/1984-0063.20200034

**Published:** 2021

**Authors:** Gabriele Ramos de Luccas, Giédre Berretin-Felix

**Affiliations:** 1 University of São Paulo’s Bauru School of Dentistry, Department of Speech-Language Pathology and Audiology -Bauru - SP - Brazil.

**Keywords:** Sleep Apnea, Obstructive, Deglutition Disorders, Speech, Language and Hearing Sciences

## Abstract

Patients with obstructive sleep apnea (OSA) may show signs and symptoms of altered swallowing function since repetitive episodes of OSA may cause hypoxia (decreased oxygen concentration in the blood) and hypercapnia (increased carbon dioxide concentration in the blood), as well as neuromuscular changes in the tissues involved, including the pharynx. This study aims to analyze whether patients with OSA show signs and symptoms of altered swallowing function. A literature search was performed in the PubMed, LILACS, Medline, Scopus, and SciELO databases by using the following search strategy: (“dysphagia”) or (“deglutition disorders”) and (“obstructive sleep apnea”) or (“obstructive sleep apnea syndrome”). The included articles were sorted by authors, year, country, journal of publication, and type of study, as well as analyzed according to the objectives, case series, OSA and swallowing assessment methods, results and conclusions. After applying the inclusion and exclusion criteria, ten articles published in medicine, dentistry and physiology journals between 1999 and 2018 were selected. The analysis of the selected articles showed that the number of participants and group structuring vary according to the study and instrumental and objective exams are used to assess swallowing and sleep in most articles. Also, the results show that groups of patients with OSA can present altered swallowing reflex, altered latency time and inspiratory suppression time, and presence of premature posterior escape, residues, penetration and aspiration. Premature posterior escape was the most frequent sign found. In conclusion, patients with OSA may show signs and symptoms of altered swallowing function.

## INTRODUCTION

Obstructive sleep apnea (OSA) involves upper airway obstruction that causes respiratory arrests during sleep^1^. It has been considered a public health issue due to its high prevalence^2^, morbidity^3^ and mortality^4^. Studies in several medical and related fields aim to improve diagnostic methods and analyze the effectiveness of treatment proposals for OSA. So far, there have been favorable and promising results in several specialties^5,6,7^.

Besides the progress in assessment and intervention methods, multidisciplinary team health professionals are increasingly interested in doing further research on the possible consequences of the pathophysiological process of OSA that could affect these patients’ performance in daily life activities and quality of life.

Within this context, the feeding of patients with OSA – especially regarding their swallowing function – represents an important study scope. Swallowing is a complex function whose main objective is to transport food from the mouth to the stomach to ensure proper body nutrition and hydration^8^. Neurological^9,10^, mechanical^11,12^, aging- related (presbyphagia)^13^ and psychogenic factors^14^, among others, can compromise the swallowing process and cause signs and symptoms during feeding such as choking, cough, throat clearing, and sensation of food stuck in the throat. Besides, it can also jeopardize the patient’s nutrition and hydration.

Histological studies show that repetitive episodes of sleep apnea can cause neuromuscular damage to tissues^15,16^. As the pharynx plays an important role in the process of swallowing by helping to protect the airways and ensuring the transport of the bolus to the esophagus without residues remaining^17^, patients with OSA may present signs and symptoms of altered swallowing function. Besides that, the presence of impaired swallowing reflex, changes in gas exchange, altered orofacial sensibility, and anatomical factors in these patients can contribute to that association.

For that reason, this study aims to analyze whether patients with OSA show signs and symptoms of altered swallowing function.

## MATERIAL AND METHODS

This is an integrative literature review with a descriptive, critical approach. A literature search was performed in the PubMed, LILACS, Medline, Scopus and SciELO databases by using the following search strategy: (“dysphagia”) or (“deglutition disorders”) and (“obstructive sleep apnea”) or (“obstructive sleep apnea syndrome”). The search was also performed by using the corresponding terms in Portuguese, and the latest database search was performed in October 2019.

The search included scientific articles in Portuguese or English, with no time limit, which aimed to investigate signs and symptoms of dysphagia in patients with OSA. Articles that evaluated swallowing by using instrumental exams, clinical protocols, or symptom questionnaires were included, as well as those with instrumental exams during sleep to record OSA episodes.

Papers that were not fully available by using a Virtual Private Network (VPN) system were excluded, as well as other review studies and research aimed both to investigate how surgical treatments for OSA impact the swallowing function and to understand the swallowing of patients with OSA during sleep. Also, articles that included patients with neurological diseases, head and neck cancer, previous radiotherapy, gastroesophageal diseases, psychiatric disorders, and who had used sedative drugs were not considered. Besides, the authors reviewed the references of the selected articles to include papers that were not found through the search strategies mentioned.

Initially, the authors analyzed the database articles’ title and abstract so they could apply the inclusion and exclusion norms and later select whichever met such criteria. A full reading of the selected articles was then performed to prove that they met the research objectives. Finally, the included articles were sorted by authors, year, country, journal of publication, and type of study, as well as analyzed according to the objectives, case series, OSA and swallowing assessment methods, results and conclusions. Regarding the findings of instrumental swallowing tests, latency time was defined as the period between bolus ejection and onset of swallowing; inspiratory suppression time is the time from swallowing termination to the next onset of inspiration^18^; premature posterior escape means the premature escape of the bolus into the hypopharynx, going further than where the pharyngeal response should occur; presence of residues means the bolus remained in the pharynx, vallecula and pyriform recesses after swallowing; penetration means the bolus enters the airway but not below the true vocal folds; and aspiration is the passing of the bolus below the true vocal folds^19^.

## RESULTS

**Figure 1:**
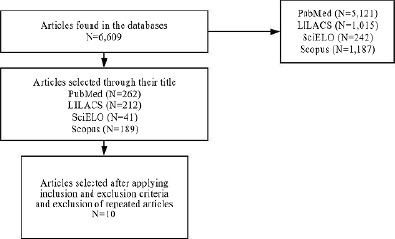
Flowchart of the article selection process.

The flowchart presented in Figure 1 shows the process of selecting articles in the databases according to search strategies. Ten articles were selected after applying the inclusion and exclusion criteria.

Table 1 shows these articles in chronological order of publication according to their authors, year, country and journal of publication, and type of study. The publication period of the articles was between 1999 and 2018, mostly in international journals in the fields of medicine, dentistry and physiology. Brazil and Japan published the largest number of articles in the field, and most of them are cross-sectional and prospective.

**Table 1:** Articles according to their authors, year, country and journal of publication, and type of study.

Article	Authors	Education	Year	Country	Journal	Type of Study
1^18^	Teramoto, Sudo, Matsuse, Ohga, Ishii, Ouchi et al		1999	Japan	Chest	Cross-sectional and prospective
2^20^	Teramoto; Ishii; Matsuse		2001	Japan	Dysphagia	Cross-sectional and prospective
3^21^	Jaghagen; Franklin; Isberg		2003	Sweden	Dentomaxillofacial Radiology	Cross-sectional and prospective
4^22^	Jobin, Champagne, Beauregard, Charbonneau, McFarland, Kimoff		2007	Canada	Journal of Applied Physiology	Cross-sectional and prospective
5^23^	Valbuza, Oliveira, Zancanella, Conti, Prado, Carvalho et al		2011	Brazil	Sleep Breath	Cross-sectional and prospective
6^24^	Schindler, Mozzanica,Sonzini, Plebani, Urbani, Pecis et al		2014	Italy	Sleep Breath	Cross-sectional and prospective
7^25^	Oliveira; Fontes; Cahali		2015	Brazil	Brazilian Journal of Otorhinolaryngology	Cross-sectional and prospective
8^26^	Wang, Li, Lee, Shieh, Lin		2016	Taiwan	Dysphagia	Cohort
9^27^	Kato, Abe, Mikami, Sugita, Muraki, Okura et al		2016	Japan	The Journal of Craniomandibular & Sleep Practice	Cross-sectional and prospective
10^28^	Valarelli, Corradi, Grechi, Eckeli, Aragon, Küpper et al		2018	Brazil	Journal of Oral Rehabilitation	Cross-sectional and prospective

Table 2 shows the objectives, case series, OSA and swallowing assessment methods, results and conclusion of each of the included articles. Their objectives include identification and analysis of signs and symptoms of swallowing disorders in patients with OSA. The number of participants and group structure vary according to the study. Objective and instrumental examinations are used to assess swallowing and sleep in most articles, and the results show that the groups of patients with OSA showed signs and symptoms of altered swallowing, such as altered swallowing reflex, latency time and inspiratory suppression time. By considering in this review patients with OSA who were assessed by using instrumental swallowing examination to analyze signs of swallowing disorders, the data show that premature posterior escape, residues, penetration, and aspiration were the signs of altered swallowing in those patients with OSA. Premature posterior escape was the most frequent sign.

**Table 2.** Objectives, case series and methods of the included articles.

Article	Objective	Case Series	OSA Assessment Methods	Swallowing Assessment Methods	Results shown by patients with OSA	Conclusion
1	To examine the relationship between the swallo+wing function and sleep breathing disorders in patients with OSA	20 patients with OSA and 20 control patients	- PSG	- Swallowing provocation test considering: latency time, respiratory suppression time, and minimum water volume required to stimulate swallowing response	- Higher latency time values (50%) - Lower inspiratory suppression time values (1.95 ± 1.03) - Higher water volume needed to stimulate swallowing (25%)	Patients with OSA are likely to have a swallowing reflex disorder probably due to impaired upper airway neural and muscle function
2	To investigate the relationship between the swallowing function and gas exchange during the day and night in patients with OSA	24 patients with OSA and 24 control patients	- PSG	- Swallowing Provocation Test considering: latency time, respiratory suppression time, and the minimum water volume required to stimulate swallowing response	- Longer latency time (2.7 ± 1.5) - Shorter inspiratory suppression time (2.0 ± 1.0) - Higher water volume required to stimulate swallowing response (0.8 ± 0.5)	Hypoxia and hypercapnia may be associated with one of the impaired swallowing function mechanisms in patients with OSA.
3	To investigate whether patients with OSA have more alterations in their swallowing function in comparison with patients who snore (with or without OSA) and with the control group of patients who do not snore.	66 patients with OSA and 15 control patients	- PSG	- Swallowing videofluoroscopy of a 2-cm piece of bread and 10 ml of water with a high-fat milk consistency	- Altered pharyngeal phase of swallowing (51%) - Patients with severe OSA showed altered pharyngeal phase of swallowing (50%) - Patients with moderate and mild OSA showed altered pharyngeal phase of swallowing (61%) - Clinical signs found: premature posterior escape (48%), pharyngeal residues (11%), and penetration (5%)	Patients who snore showed a higher risk of developing altered pharyngeal phase of swallowing, regardless of concomitant OSA
4	To determine whether the sensory alteration of upper airway mucosa contributes to altered swallowing function in patients with OSA	15 men with OSA and nine control patients	- PSG	Sensory analysis considering the criteria of vibration sensitivity threshold and two-point discrimination threshold - Swallowing Provocation Test considering: latency time, respiratory suppression time, and the minimum water volume required to stimulate swallowing response	- Impaired mucosal sensitivity - Shorter swallowing latency time (3.29 ±0.71) - Significant inverse relationship between vibration sensitivity threshold and inspiratory suppression time (r= -0.52)	Oropharyngeal sensory impairment in OSA was associated with an attenuation of inhibitory modulators to reflex control and central control of the upper airway swallowing function.
5	To show the swallowing function in patients with OSA through nasal fibroendoscopy	11 patients with OSA and 14 control patients.	- PSG	Swallowing nasal fibroscopy using 5 ml and 10 ml solid, purée, and thin liquid consistencies.	- Clinical signs found: premature posterior escape (64%) pharyngeal and vallecular residues (55%) - No cases of penetration and aspiration The risk of altered swallowing did not increase after an increased severity of OSA.	Patients with OSA showed clinical manifestations of altered swallowing when assessed through nasal fibroscopy.
6	To analyze the signs and symptoms of oropharyngeal dysphagia in patients with OSA	72 patients with OSA	- PSG	Nasofibroscopic assessment of swallowing by offering 5, 10, and 20 ml of liquid and purée and ¼ of an 8 g cookie - Penetration-Aspiration Scale (PAS) - Residue scale - DOSS scale - SWAL-QOL quality-of-life questionnaire	- Clinical signs found: premature posterior escape (64%); multiple swallows (28%), penetration (35%), aspiration (3%) residue (44%) - Patients with severe OSA scored higher on the SWAL-QOL food selection subscales	Patients with OSA showed altered swallowing function; however, the severity of such dysfunction was not correlated with the severity of OSA
7	To search for altered pharyngoesophageal manometry in patients with obstructive sleep apnea with and without oropharyngeal dysphagia	22 patients with OSA	- PSG - Epworth Sleepiness Scale	- Dysphagia Symptom Questionnaire - Swallowing nasofibroscopic - Pharyngoesophageal manometry with measurements of lower and upper esophageal sphincter pressure and mean pharyngeal pressure at three levels during swallowing	- Swallowing symptoms on the questionnaire (9.1%) - Altered swallowing on the swallowing nasofibroscopic (45.5%) - Swallowing symptoms and altered swallowing nasofibroscopic (22.7%) - Clinical signs found: premature posterior escape (45.5%), pharyngeal residues (22.7%), penetration (13.6%) There was no statistically significant difference between patients with or without dysphagia regarding manometry measurements, age, and severity of OSA	Pharyngoesophageal manometry showed no significant difference between the groups with and without oropharyngeal dysphagia
8	To investigate swallowing and its coordination with breathing in patients with OSA	39 men with OSA and 35 control patients	-PSG	- Electrophysiological monitoring system comprising electrodes and nasal cannula. Each patient was instructed to swallow volumes of 1, 3, 5, 10, and 20 ml of water.	- Longer duration of total laryngeal excursion and shorter duration of submental muscle contraction - Longer breathing pause for swallowing - Significantly more fragmented swallowing when swallowing volumes of 10 and 20 ml of water	Altered coordination between breathing and swallowing in patients with OSA.
9	To investigate the prevalence of clinical symptoms related to altered swallowing in a sample of patients with OSA.	507 patients with OSA	- Physical clinical examination - Questionnaire for swallowing symptoms - Nocturnal cardiorespiratory assessment	- 15-question validated questionnaire for dysphagia screening	- 16.2% had at least one of the symptoms of altered swallowing, and 6.3% had two or more symptoms - The most frequent symptom was difficulty to cough during or after meals Demographic, sleep-related characteristics, and clinical variables were not significant between patients with and without symptoms of altered swallowing	Patients with OSA reported symptoms of altered swallowing regardless of the severity of their OSA.
10	To identify OSA-related muscle, hyoid, and swallowing changes and correlate such changes with the severity of OSA	60 patients with OSA and 12 control patients	- PSG	- Cephalometry - Orofacial Myofunctional Evaluation (OMES) - Swallowing videofluoroscopy	- Lower hyoid position and narrower posterior air distance in comparison to controls - Lower scores on orofacial myofunctional assessment - Shorter hyoid and velum contraction times during videofluoroscopy - Hyoid position associated with the severity of OSA - Clinical signs found: premature posterior scape during liquid (20%) and pasty (62%), penetration (3%) - Impaired muscle pattern and swallowing regardless of the severity of OSA and facial profile	Patients with OSA have a higher predisposition to inferior hyoid bone positioning, as well as orofacial and swallowing myofunctional disorders

PSG = Polysomnography; OSA = Obstructive sleep apnea.

## DISCUSSION

This literature review aimed to discover whether patients with OSA show signs and symptoms of dysphagia. At the end of the search, ten scientific articles were identified in the databases.

The information and results presented show that most articles (six) were published in the lasts, which highlights the recent tendency of studies that try to understand the several consequences of OSA in the individual as a whole. The samples varied in the studies presented, and seven pieces of research had control groups with healthy individuals, which is an important methodological strategy to allow the comparison of evaluation data with the experimental group, thus understanding the results more effectively^29^.

Most methods to diagnose OSA used polysomnography, which is critical since it is considered the gold standard for the diagnosis of sleep disorders^30^. Of the ten articles analyzed in this review, nine used at least one method of instrumental assessment to assess the swallowing function, such as videofluoroscopy, nasal fibroendoscopy, pharyngoesophageal manometry and swallowing provocation test. Using instrumental assessment methods is important to analyze patients in an objective and standardized way, and it also makes it possible to avoid research biases resulting from subjective clinical analysis of evaluators and reports of the patients themselves obtained through questionnaires^31,32^. On the other hand, although instrumental examinations are the gold standard for assessing altered swallowing function, it is key to analyze the effectiveness of clinical assessments and screenings as a way to identify patients with possible alterations in such function on a larger scale through methods that are both simpler and of easier access for health professionals, since instrumental assessments are available in few centers.

Regarding signs and symptoms of dysphagia, all the articles presented found altered swallowing function in patients with OSA, mainly as altered swallowing reflex, altered latency time and inspiratory suppression, and presence of premature posterior escape, residues, penetration and aspiration. The pathogenesis of OSA causes neuromuscular and sensory alterations in the upper airway tissues^33^, which can cause damage to the protection of the airway during swallowing and swallowing efficiency.

Premature posterior escape was the most frequent sign found in patients with OSA, which is nor mally associated with an inefficiency of the oral phase of swallowing. The orofacial structures, such as lips, tongue and cheeks are important to guide the bolus to the pharynx, and if there are any alterations in mobility, tonicity and sensitivity of these structures, there may be damage to the bolus organization and ejection, causing the presence of altered pharyngeal phase of swallowing, such as premature posterior escape^34^. As evidenced in some studies, patients with OSA may present altered aspects of orofacial mobility and tonicity, which may justify this finding^35,36,37^.

Since both premature posterior escape and residues are commonly related to difficulty in oral motor control and tongue function^38^, these parameters are important to be investigated in this population. Except for the study by Valarelli et al.^28^, the studies presented did not assess the patients’ oral myofunctional aspects, suggesting there is a need for further studies focused on this such aspects to better understand the functioning of the oral and pharyngeal swallowing phases in patients with OSA.

The presence of penetration may be related to incoordination between both swallowing and breathing functions, altered swallowing reflex, decreased pharyngeal sensitivity and hyoid bone position. The authors of the articles already highlighted these associations^22,26,28^. As shown in one of the studies, a low occurrence of aspiration may be related to longer latent time seen in these patients, which would also lead to individuals with altered swallowing function despite having no symptoms^24^.

The articles included for analysis have a heterogeneous methodology. The number of participants in each study varies, and not all of them have a control group, which explains the statistical differences in the occurrence of certain clinical signs in the articles. Besides, some studies did not consider different consistencies and volumes of food in the swallowing assessment, which makes it difficult to discuss the clinical signs found and their relationship with viscosity and volume.

Considering the knowledge gaps this review found in the articles, we suggest new studies to assess swallowing in patients with OSA. These should consider different consistencies and volumes of food to investigate the possible variability in clinical signs and groups with different age ranges to understand the effect of aging on the swallowing function in patients with OSA.

Regarding the study designs it is worth stressing that most articles presented here (nine) have a cross-sectional design. That means patient assessment was performed at a single moment, so it is not possible to establish a cause-effect relationship between OSA and dysphagia, and it is only possible to indicate an important association. Further cohort studies are suggested to understand if this relationship exists as this design allows longitudinal follow-up of a group of subjects (patients with OSA) to identify a risk factor (occurrence of dysphagia).

The articles included in this review have been published mainly in medicine, dentistry and physiology-focused journals, and only one of the studies was performed by speech-language therapists. The interface between speech-language pathology and sleep medicine is established mainly through myofunctional therapy^39^, and randomized controlled trials have shown promising and positive results in reducing the signs and symptoms in patients with OSA and in association with other treatment methods^35,36,37,40^.

Within the context of speech-language therapy, dysphagia is one of these professionals’ specialties, who perform clinical – and, if possible, instrumental – assessment of oropharyngeal swallowing to identify possible altered functions. These clinicians also perform speech therapy aimed to rehabilitate swallowing through specific exercises and techniques^41^. Therefore, considering the relationship between obstructive sleep apnea and altered swallowing function, research development by those professionals is key to define the best diagnostic methods for those patients, and mainly to discuss the introduction of exercises and intervention strategies for the swallowing function in the therapeutic planning of patients with OSA who show signs and symptoms of dysphagia.

Regarding the results of the swallowing function rehabilitation in patients with OSA, no studies with that objective were found in the literature. Clinical practice in oropharyngeal dysphagia often uses oropharyngeal and vocal exercises and protective and facilitating swallowing maneuvers to eliminate or decrease signs and symptoms of oropharyngeal dysphagia. These include the presence of waste, premature escape, delay of pharyngeal phase initiation, penetration and aspiration, among others. Applying these strategies to patients with OSA to improve their swallowing function is promising. However, it is important to: a) consider the pathophysiological aspects of OSA that differ from other underlying diseases related to dysphagia; b) list the exercises and maneuvers that are most suitable to the cases; and, subsequently, c) conduct clinical trials that prove whether these strategies are effective in that population.

Based on the results by this literature review, we can conclude that patients with OSA may present signs and symptoms of dysphagia, such as altered swallowing reflex, latency time and inspiratory suppression time, and presence of premature posterior escape, residues, penetration and aspiration.
